# Motor-like DNA motion due to an ATP-hydrolyzing protein under nanoconfinement

**DOI:** 10.1038/s41598-018-28278-0

**Published:** 2018-07-03

**Authors:** Maedeh Roushan, Zubair Azad, Saeid Movahed, Paul D. Ray, Gideon I. Livshits, Shuang Fang Lim, Keith R. Weninger, Robert Riehn

**Affiliations:** 1 Department of Physics, North Carolina State University, Raleigh, NC USA; 2 Present Address: Department of Chemistry, Osaka University, Osaka, 560-0043 Japan

## Abstract

We report that long double-stranded DNA confined to quasi-1D nanochannels undergoes superdiffusive motion under the action of the enzyme T4 DNA ligase in the presence of necessary co-factors. Inside the confined environment of the nanochannel, double-stranded DNA molecules stretch out due to self-avoiding interactions. In absence of a catalytically active enzyme, we see classical diffusion of the center of mass. However, cooperative interactions of proteins with the DNA can lead to directed motion of DNA molecules inside the nanochannel. Here we show directed motion in this configuration for three different proteins (T4 DNA ligase, MutS, *E. coli* DNA ligase) in the presence of their energetic co-factors (ATP, NAD^+^).

## Introduction

The statistical mechanics of DNA confined to long and narrow nanofluidic channels have been explored extensively, and an increasingly complete picture is emerging^1–6^. For the purpose of this paper, we only consider quasi-onedimensional channels with a cross-section on the order of 100 × 100 nm^2^, and tens to hundreds of microns long. In these nanochannels, DNA is stretched out to yield a constant stored contour length of DNA per unit distance along the channel axis. The mapping of genetic landmarks on nanochannel-stretched DNA has become sufficiently mature to meaningfully contribute to genome science at large^7,8^. Epigenetic profiling is emergent^9–12^.

The stretching technique has been extended from DNA to DNA-protein complexes, and the analysis of compaction or extension of nucleic acids by DNA-binding proteins has been developed into an analytic technique with considerable power^13–17^. The majority of studies of DNA-protein interactions are strictly in equilibrium, and even studies that are not in equilibrium are performed in a regime where reversibility can reasonably be assumed^18^.

However, this assumption of reversibility does not hold for protein-DNA interactions where enzymatic activity coupled with modification of covalent chemical bonds is a key feature. For instance, the activity of endonucleases is profoundly irreversible, and has been monitored within nanochannels^19,20^. It appears though that endonucleases are not a good model for a general DNA-modifying enzyme because the energy source is the DNA itself. In contrast, wide classes of DNA-modifying enzymes such as helicases, isotopomerases and ligases utilize adenosine triphosphate (ATP) or a similar energetic compound to establish the preferred direction of the reaction cascade^21^.

In this publication, we present a detailed test of one such enzyme, T4 DNA ligase, and an exploratory overview of others. We find that the catalytic activity is linked to a superdiffusive displacement of the DNA molecule within the nanochannel that is consistent with a constant mean velocity for each molecule. We proceed to propose a speculative model for the superdiffusive motion. While the motion superficially resembles that of a protein filament transported by a set of surface-tethered proteins^22–24^, we argue that a motor-like action in that narrow sense is not present here. Instead, we propose that the transport effect is due to self-maintaining concentration polarization of ions within the channel.

## Results

**Figure 1:**
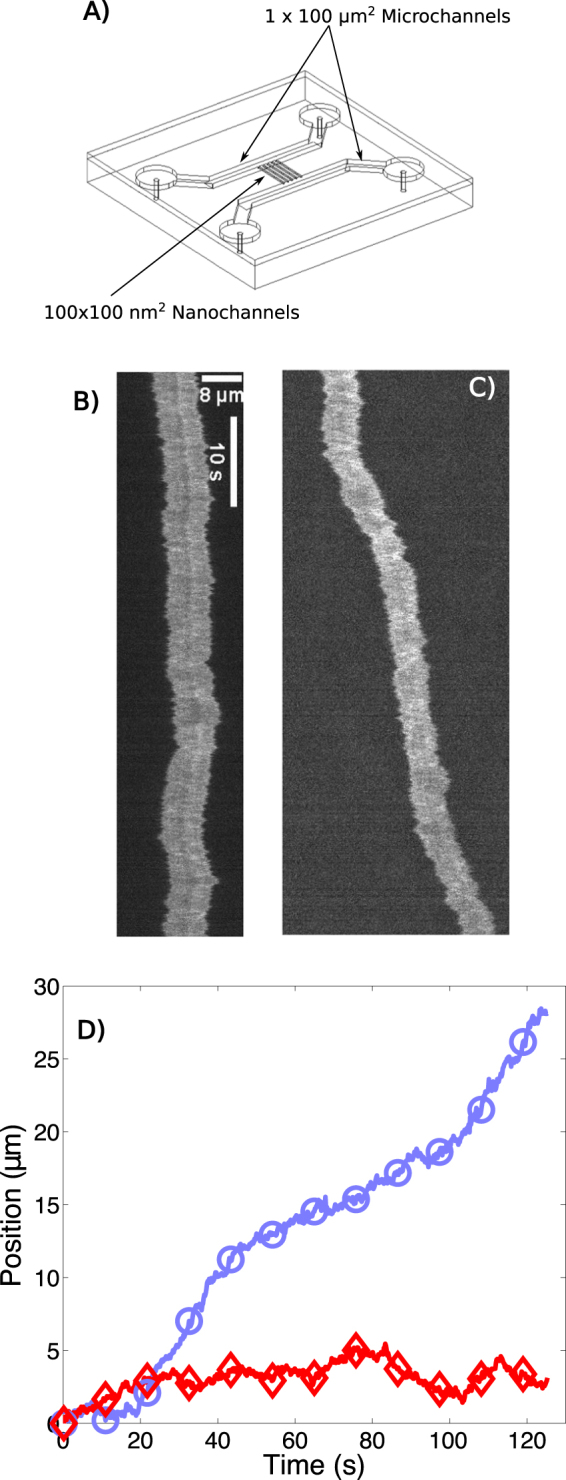
(**A**) Schematic of the device. (**B,C**) Kymographs showing the fluorescent intensity along nanochannel axis as a function of time for bare *λ*-DNA (**B**) and *λ*-DNA with T4 DNA ligase in a catalytically active buffer (**C**), respectively. (**D**) Center of mass of the molecules in (**B**) (red, diamonds) and (**C**) (blue, circles) as function of time.

Experiments were performed with fluorescently stained *λ*-DNA in Tris-Borate (TB) buffer. The buffer was supplemented with T4 DNA ligase, ATP, MgCl_2_ and EDTA if required. The sample was introduced into large microchannels (1 × 100 *μm*^2^ cross-section, 1 *cm* long) of the fused silica device (Fig. 1A), and then driven into the nanochannels (100 × 100 *nm*^2^ cross-section, 200 *μm* long) using pressure. The pressure was appropriately low such that the majority of molecules were introduced in a linear configuration^25^, and any folded molecules were discarded.

Figure 1B shows a kymograph of the intensity along a nanochannel axis after a single *λ*-DNA molecule was introduced and the pressure was removed. No T4 DNA ligase, Mg^2+^, or ATP was present. We observe the commonly reported intensity and length fluctuations linked to internal fluctuations, and an apparently random displacement of the center of mass of the molecule. Figure 1C shows the outcome of the same experimental procedure, but with T4 DNA ligase, Mg^2+^, and ATP present. DNA appears somewhat less extended - an effect previously attributed to the presence of ATP^26^ (see Supp. Mat. C for length analysis). While this molecule undergoes the same internal and length fluctuations as the molecule in Fig. 1B, its center of mass mass appears to drift into one direction.

We determined the center of mass and extension for each video frame by fitting the brightness along the molecule backbone to the convolution of a Gaussian and a boxcar function^1^. In Fig. 1D, we compare the center of mass position for the two molecules presented in Fig. 1B and Fig. 1C. This analysis was performed for all molecules of the set, and we find in general that DNA with catalytically active T4 DNA ligase (Fig. 1C) undergoes directed motion. The motion appears locally piecewise linear.

**Figure 2:**
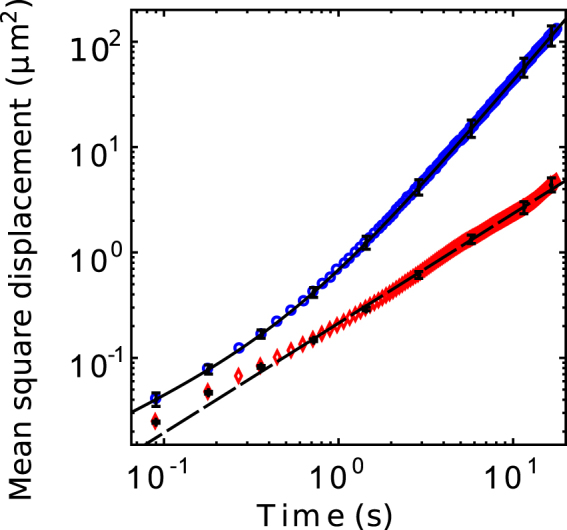
Average mean-square displacement curves calculated from the center of mass position for 20 molecules for each condition. Blue circles correspond to data DNA with T4 DNA ligase in the presence of its cofactors, while red diamonds indicate bare DNA. Error bars are the standard deviation between molecules of the experimental set, and are depicted only for select data points to illustrate the trend. The continuous and dashed curves correspond to the fits for the conditions as described in the text.

The natural question arising is whether the motion under protein action can be described by a directed translocation, a classical diffusion with increased diffusion coefficient (when compared to DNA without any cofactors), or an entirely different process. To answer this question, we have prepared mean square displacement (MSD) graphs for both DNA without T4 DNA ligase or co-factors and with T4 DNA ligase and co-factors over a set of 20 molecules for each condition (Fig. 2). If the motion were governed by classical diffusion, we would anticipate that the MSD grows linearly with time, i.e. $\langle {\rm{\Delta }}{x}^{2}({\rm{\Delta }}{t})\rangle \propto {\rm{\Delta }}{{t}}^{{\alpha }}$ with *α* = 1. If it grows as a power law with $\alpha  > 1$ we call the motion “superdiffusive”, and if *α* = 2 we have a the special case of directed translocation in a strict sense with a well-defined mean speed.

The graph for protein-free DNA in Fig. 2 shows a well defined single-power-law relationship $\langle {\rm{\Delta }}{x}^{2}({\rm{\Delta }}t)\rangle =$ $\mathrm{(0.21}\pm \mathrm{0.01)}\mu {m}^{2}{({\rm{\Delta }}t\frac{1}{s})}^{1.04\pm 0.01}$ (error is the 95% confidence interval of the fit). The curve is evidently consistent with classical diffusion, except for a mildly subdiffusive regime at very small times that likely is caused by fitting errors. The MSD curve for DNA with catalytically active T4 DNA ligase clearly shows superdiffusive behavior. We find a good fit to the data by using a double-power law of the form $\langle {\rm{\Delta }}{x}^{2}({\rm{\Delta }}t)\rangle =\mathrm{(0.23}\pm \mathrm{0.01)}\mu {m}^{2}$ ${({\rm{\Delta }}t\frac{1}{s})}^{0.77\pm 0.01}+\mathrm{(0.45}\pm \mathrm{0.01)}\mu {m}^{2}{({\rm{\Delta }}t\frac{1}{s})}^{1.96\pm 0.01}$, indicating translocation at constant speed of 0.7 *μm*/*s* for long times, and a mildly subdiffusive regime at very short times. For times between 1 *s* and 15 *s*, the motion is very well described by directed translocation in a single direction.

For finding a working hypothesis for the translocation of DNA in presence of catalytically active T4 DNA ligase, we first evaluated whether the relatively complex buffer system could be a source of the motion (Table 1 and Supp. Mat. D.i.). We found that all of the buffer components alone, as well as combinations of components, did not induce a superdiffusive motion of DNA within the nanochannel. We then evaluated whether the presence of the protein itself could be a source of translocation. We tested T4 DNA ligase without any co-factors, with only ATP (as in a prior publication^18^), with only Mg^2+^ and EDTA, and with the full buffer system as described above. Only the full system supported translocation. We further note that using human genomic DNA also gave rise to the directed translocation, indicating that the motion is not substrate-specific.

**Table 1:** Survey of different buffer conditions and under action of different proteins.

Solution	Drift	Best fit for ${\boldsymbol{\langle }}{{\boldsymbol{x}}}^{{\bf{2}}}{\boldsymbol{(}}{\boldsymbol{\Delta }}{\boldsymbol{t}}{\boldsymbol{)}}{\boldsymbol{\rangle }}$
Bare *λ*-DNA	No	$0.21\,\mu {m}^{2}{({\rm{\Delta }}t\frac{1}{s})}^{1.04}$
*λ*-DNA + ATP	No	$0.20\,\mu {m}^{2}{({\rm{\Delta }}t\frac{1}{s})}^{1.03}$
*λ*-DNA + ATP + Mg^2+^ + EDTA	No	$0.22\,\mu {m}^{2}{({\rm{\Delta }}t\frac{1}{s})}^{0.94}$
*λ*-DNA + T4 DNA ligase	No	$0.56\,\mu {m}^{2}{({\rm{\Delta }}t\frac{1}{s})}^{0.77}$
*λ*-DNA + T4 DNA ligase + ATP	No	$0.18\,\mu {m}^{2}{({\rm{\Delta }}t\frac{1}{s})}^{0.96}$
*λ*-DNA + T4 DNA ligase + Mg^2+^ + EDTA	No	$0.48\,\mu {m}^{2}{({\rm{\Delta }}t\frac{1}{s})}^{0.80}$
*λ*-DNA + T4 DNA ligase + ATP + Mg^2+^ + EDTA	Yes	$0.23\,\mu {m}^{2}{({\rm{\Delta }}t\frac{1}{s})}^{0.77}+0.45\,\mu {m}^{2}{({\rm{\Delta }}t\frac{1}{s})}^{1.96}$
*λ*-DNA + T4 DNA ligase + ATP + Mg^2+^ + EDTA + BSA	No	$0.38\,\mu {m}^{2}{({\rm{\Delta }}t\frac{1}{s})}^{0.92}$
*λ*-DNA + E-coli DNA ligase + NAD^+^ + Mg^2+^ + EDTA	Yes	$0.17\mu {m}^{2}\,{({\rm{\Delta }}t\frac{1}{s})}^{0.46}+0.16\mu {m}^{2}{({\rm{\Delta }}t\frac{1}{s})}^{1.8}$
*λ*-DNA + E-coli DNA ligase + ATP + Mg ^2 +^ + EDTA	No	$0.07\,\mu {m}^{2}{({\rm{\Delta }}t\frac{1}{s})}^{0.19}+0.14\,\mu {m}^{2}{({\rm{\Delta }}t\frac{1}{s})}^{1.03}$
*λ*-DNA + MutS + Mg^2+^ + EDTA	No	$0.11\,\mu {m}^{2}{({\rm{\Delta }}t\frac{1}{s})}^{0.79}$
*λ*-DNA + MutS + ATP + Mg^2+^ + EDTA	Yes	$0.21\,\mu {m}^{2}{({\rm{\Delta }}t\frac{1}{s})}^{0.44}+0.49\,\mu {m}^{2}{({\rm{\Delta }}t\frac{1}{s})}^{1.95}$
*λ*-DNA + MutS + *γ*-ATP + Mg^2+^ + EDTA	Minimal	$0.07\,\mu {m}^{2}{({\rm{\Delta }}t\frac{1}{s})}^{0.6223}+0.03\,\mu {m}^{2}{({\rm{\Delta }}t\frac{1}{s})}^{1.68}$

Errors as determined by the 95% confidence interval of fit are at or below significant digits. MutS was used at 200 *nM* and the same conditions as T4 DNA ligase, except for that ATP was replaced by *γ*-ATP for one set. *E. coli* DNA ligase (New England Biolabs) was used at 0 8 *kU*/*ml* either in the same buffer as used for T4 DNA ligase, or in the supplier-provided Tris-based buffer that contained 26 *μM* NAD^+^ instead of ATP and was supplemented with 2 mM EDTA. Curves underlying the fits are presented in the supplementary materials section D.

It would seem that DNA is transported in a fashion superficially similar to a system in which motor proteins are coupled to the surface of a channel with sub-persistence length width in which a filament is transported on its surface^24^. A preliminary, and very unlikely hypothesis thus would require T4 DNA ligase to have the action of a motor protein. While T4 DNA ligase is a widely-used “workhorse” enzyme, it is not as well-studied as T7 DNA ligase^27^. Its known functions include blunt-ended ligation, ligation coupled to hybridization, lyase activity, nick-sealing, gapped-ligase, and single-stranded ligation^28–32^. The influence of adenylation of T4 DNA ligase (through ATP) and the impact of Mg^2+^ on binding and catalytic activity have been studied^33–35^. However, under no circumstance has a motor-like search mechanism coupled to ATP hydrolysis been even suggested. We thus decided to test whether two other DNA-binding proteins with known catalytic activity coupled to an energetic compound could cause the directed motion.

*E. coli* DNA ligase is an interesting alternative to T4 DNA ligase as it utilizes NAD^+^ instead of ATP for adenylation^36^. It also likely has fewer functions than T4 DNA ligase and thus presents a “cleaner” system. Using *λ*-DNA and the manufacturer-supplied Tris-based buffer supplemented with 2 *mM* ATP, we find that *E. coli* DNA ligase does catalyze translocation in presence of NAD^+^ with a close-to linear displacement versus time relationship but about half the translocation speed observed using T4 DNA ligase (Table 1 and Supp. Mat. D.iii.). In a buffer that contained ATP instead of NAD^+^, double-power law curve was also required to fit the MSD versus time graph, but even the faster of the two components merely had *α* = 1.03 indicating classical diffusion and not directed translocation (Table 1 and Supp. Mat. D.iii.). Thus, the translocation appears distinctively linked to the specific interaction of an energetic compound and protein, and not some unspecific interaction of ATP with protein and DNA.

MutS is an extensively characterized protein that is part of the mismatch recognition and repair pathway with an ATPase domain^37,38^. We repeated the experiment with the same buffer set and substrate as described for T4 DNA ligase, and observed the characteristic directed displacement of DNA due to MutS with a drift velocity similar to the achieved by T4 DNA ligase (Table 1 and Supp. Mat. D.ii.). Using MutS in a buffer containing Mg^2+^ and EDTA but no ATP did show classical diffusion. It is established that MutS cannot utilize *γ*-ATP efficiently. We repeated the experiments with *γ*-ATP instead of ATP, and found strongly retarded directional motion with a dislocation in 10 *s* more than an order of magnitude lower than with ATP. Since MutS certainly does not contain a motor protein domain, we strongly believe that the translocation of DNA in presence of T4 DNA ligase is also not due to motor protein action.

## Discussion

Since we have established that the motion is unlikely due to a distinctive “motor action”, we must propose a mechanism that only employs the interactions of liquid and DNA, mediated by the ions in solution.

We note that the symmetry along the channel axis must be broken, either through composition of the solution, the DNA, or the protein distribution along the channel or the DNA. Furthermore, we have to identify a mechanism that propels the DNA while maintaining the asymmetry along the channel axis. In our case, possible candidates for the asymmetry is the liquid flow during insertion of the DNA molecule into the nanochannel in the form of a concentration polarization of ions, or and stochastic asymmetry in protein concentration of density of nicks along the DNA substrate. Such concentration polarization is well established at nanopore entrances^39^, and anticipated around DNA molecules^40^. In particular, ATP, AMP, and PPi will establish a non-homogeneous concentration profile. Once a concentration polarization is established, we follow observation by Pallaci *et al*. that DNA drifts into the direction of the higher ionic strength^41^. For colloidal particles the effect is well established together with a well-accepted model^42,43^, and theoretical description for DNA drift been given by Hsu^44^. A possible complication here is that of strong confinement of DNA, but we note that our experiment is similar to nanopore experiments in that aspect^45^. In the supplementary materials (section H) we summarize our experiments in which we placed nanochannel-stretched DNA into an ionic strength gradient with stationary liquid, and observed that DNA indeed drifts towards the high ionic strength nanochannel entrance. Thus, we propose that an imbalance of ionic strength between the termini of the DNA is responsible for the apparent drift.

**Figure 3:**
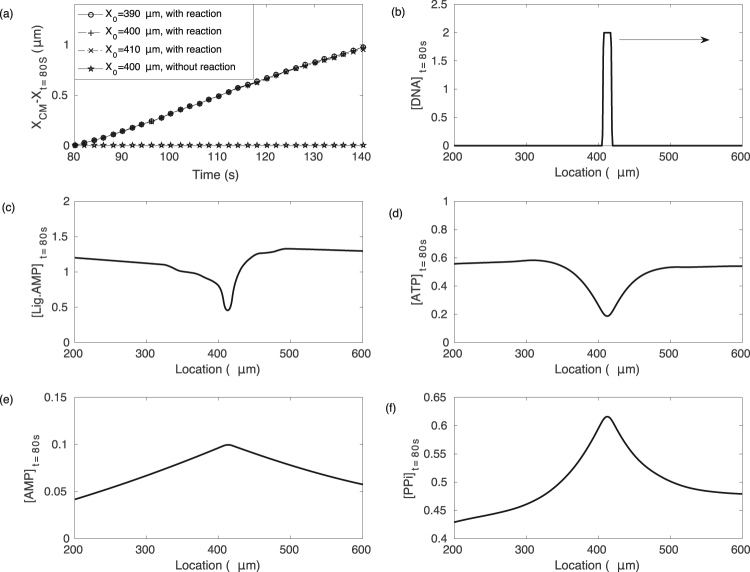
Numerical model of DNA drift under ATP hydrolysis. (**a**) DNA position versus time for multiple start positions after initial transient, as well as for model without ATP hydrolysis. (**b–f**) Concentrations of critical species in the proposed model after the steady state attained.

The imbalance in ionic strength has to be maintained by the enzymatic reaction of T4 DNA ligase. The obvious overall process that changes the ionic strength is the consumption of ATP to form AMP and free pyrophosphate (PPi) that is coupled to nick-sealing^34,35^. We propose here that under the prevailing conditions (low DNA concentration, incubation of T4 DNA ligase in ATP and Mg^2+^), the majority of T4 DNA ligase is in an adenylated state. Our experiment proceeds in a fluorescence microscope where DNA nicking is present at all times^46^, and thus a strong background rate of nick-sealing events should be anticipated. Thus, when the adenylated enzyme encounters a nick site, we propose that that AMP is released, thereby locally raising the ionic strength. The analogue leaving groups to PPi and AMP for the *E. coli* DNA ligase are *β*-nicotinamide mononucleotide (*β*-NMN) and AMP, while the leaving groups for MutS would be ADP and phosphate. This release of reaction products and linked increase of ionic strength would be occurring on the end of DNA that encounters the higher fraction of ligases poised for activity. DNA then would drift towards that terminus, enhancing the fraction of ligases ready for catalytic activity, and thus sustaining the reaction and the drift. The self-consistency of the model was numerically verified (Supp. Mat. I.), and the drift dynamics as well as the ionic profiles around DNA are are show Fig. 3. Note that removal of catalytic activity without any other modification of the model lead to complete ceasing of the drift.

In the description of the data in Fig. 1 we pointed out that the motion appeared piecewise linear in presence of T4 DNA ligase and co-factors. We analyzed this by fitting a the minimal set of piecewise linear functions to the position versus time graphs. The number of fitted segments to a kymograph was chosen such that the average squared error between fit and data became smaller than the expected error of a linear fit to purely diffusing DNA (examples and statistics shown in Supp. Mat. E). We obtain segments on average 12 seconds in duration, about the same time as covered by Fig. 2.

**Figure 4:**
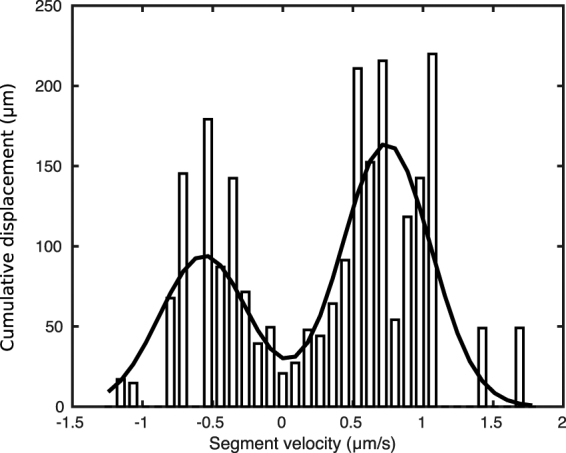
Distribution of combined displacement as function of velocity for 20 molecules. The velocity for each molecule inside the channel was calculated from the displacement versus time graphs after segmentation step illustrated in the Supp. Mat. E.

The displacement probability density for a set of 20 DNA molecules presence of catalytically active T4 DNA ligase as function of drift velocity (slope in position versus time plot) is shown in Fig. 4. The histogram is dominated by two maxima at 0.7 ± 0.1 *μm*/*s* in the forward/backward direction with a distribution half-width of 0.22 ± 0.07 *μm*/*s*. The coincidence of the maximum likely drift speeds in Fig. 4 (forward/backward peak location) and Fig. 2 (drift speed of quadratic component) strengthens our initial interpretation that the motion can be viewed as translocation with constant speed at times up to roughly 10 s. At times longer than that, Fig. 2 has insufficient statistical weight. The long-term behavior is thus best characterized by the transitions between segments the underlie Fig. 4. We categorized segments into forward, stationary, backward by setting thresholds at ±0.2 *μm*/*s*, and found that no molecule contained both forward and backward motion. We found that the majority of transitions (32/55) between a moving state and a temporary stall, and the rest contained changes of speed while keeping direction (23/55). Note that an equal number of molecules in Fig. 4 were introduced from both the left and the right. If the entrance direction was taken into account, we observed that the majority of molecules travelled into the direction of introduction.

The robustness of the drift direction raises the question whether it is imprinted to the pair or molecule and channel in some fashion. We probed this by applying ultra-low pressure (<0.1 psi) to push back the stretched DNA against its direction of travel before releasing it. We found that molecules continue to travel in the direction that was initially observed (Supp. Mat. F).

This then suggests that the temporary stall events are due to short times during which the DNA is retarded by temporary adhesion to the channel wall. Tentative indication of this mechanism can be found in Table 1, where all conditions that contain T4 DNA ligase do not show a strict classical diffusion in the short-term limit (or overall), but are instead mildly subdiffusive with *α* < 1 as would be characteristic in a system retarded by binding traps. We note that the addition of a high concentration of bovine serum albumin (BSA, 0.6 *μg*/*ml*) suppressed the drift motion (Table 1). Typically BSA is used to reduce adhesion of proteins to channel sidewalls. Thus it appears feasible that at least part of the adenylated protein that is poised to release AMP is adsorbed onto the wall, but can currently not point to its specific role.

To conclude, we report that DNA translocates through nanofluidic channels in presence of a number of catalytically active enzymes that hydrolyze an energetic compound. We propose a kinetic model that depends on an asymmetry of ionic strengths between the termini of the molecule within the channel. We further propose that the ionic strength imbalance is maintained by the enzymatic reaction. The mechanism is specific to nanochannel confined DNA since confinement causes the macromolecule to sample a spatial zone and prevents rapid dissipation of the ionic disturbance. We propose that this mechanism may be a sensitive assay for hydrolysis of energetic compounds by a wide class of enzymes that utilize DNA as a substrate.

## Methods

### Biological Materials

*λ*-DNA (Roche) was stained with YOYO-1 (Invitrogen) at a ratio of 1 dye per 20 base pairs for 48 hours at 4 °C in 0.5× TBE buffer (pH 8). The contour length of unstained *λ*-DNA (48.5 kbp) is around 16 *μm*, which increases somewhat after staining^47^. T4 DNA ligase (Roche) had a final concentration of 40 units/*ml* in a 0.5× TBE buffer (pH 8) that was modified as follows. In order to obtain catalytically active ligase, 1 *mM* ATP (Roche) and 5 *mM* MgCl_2_ were added as T4 DNA ligase cofactors to catalytically active solutions. This solution was incubated for 1 hour at 16 °C to allow full equilibrization of Mg^2+^, ATP, and T4 DNA ligase. After this incubation the free Mg^2+^ concentration was lowered by adding 2 *mM* EDTA. This is necessary since YOYO-1 is not a stable intercalator at a free Mg^2+^ concentration of 5 *mM*. Stained DNA was added to the protein yield a final concentration of 5 *μg*/*mL* followed by stabilization for 2 hours at 25 °C. Tests using alternative DNA (human genomic DNA from Roche) and an alternative enzyme provider (New England Biolabs) were conducted to exclude possible contamination. In a subset of experiments, a high concentration (0.6 *mg*/*ml*) bovine serum albumin (BSA) was added to prevent sticking to the nanochannel walls.

### Devices and observation

All experiments used mixed micro- and nanofluidic devices made from fused silica, which were prepared by methods discussed elsewhere^48^. Nanofluidic channels with a 100 × 100 *nm*^2^ cross-section and a length of 200 *μm* were placed between microchannels (see Supp. Mat. A.). Molecules were observed using an inverted fluorescence microscope with a 100×, 1.3 N.A. oil-immersion microscope objective under illumination from a metal halide lamp, and observation by EM-CCD camera (Andor) with a frame duration of 90 *ms*.

Each DNA molecule is driven from microchannel to nanochannel by a static pressure difference of 35 psi applied over the full length of the nanochannel. After the DNA molecule has entered the nanochannel, the pressure gradient is removed and the dynamics of molecules are observed.

### Data availability statement

The datasets generated during and analysed during the current study are available from the corresponding author on reasonable request.

## Electronic supplementary material

LaTeX Supplementary File

LaTeX Supplementary File

LaTeX Supplementary File

LaTeX Supplementary File

Supplementary Materials
