# Supplementary figures and images for: Motor-like DNA motion due to an ATP-hydrolyzing protein under nanoconfinement
Source: Sci Rep. 2018 Jul 3;8:10036. doi: 10.1038/s41598-018-28278-0 (PMC6030079; doi:10.1038/s41598-018-28278-0)

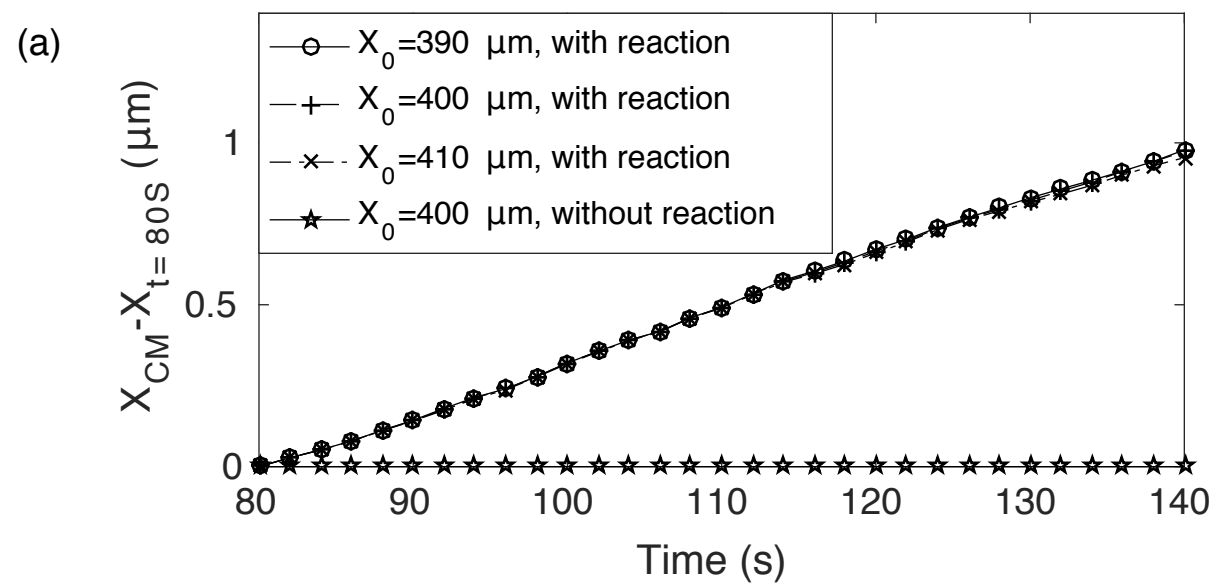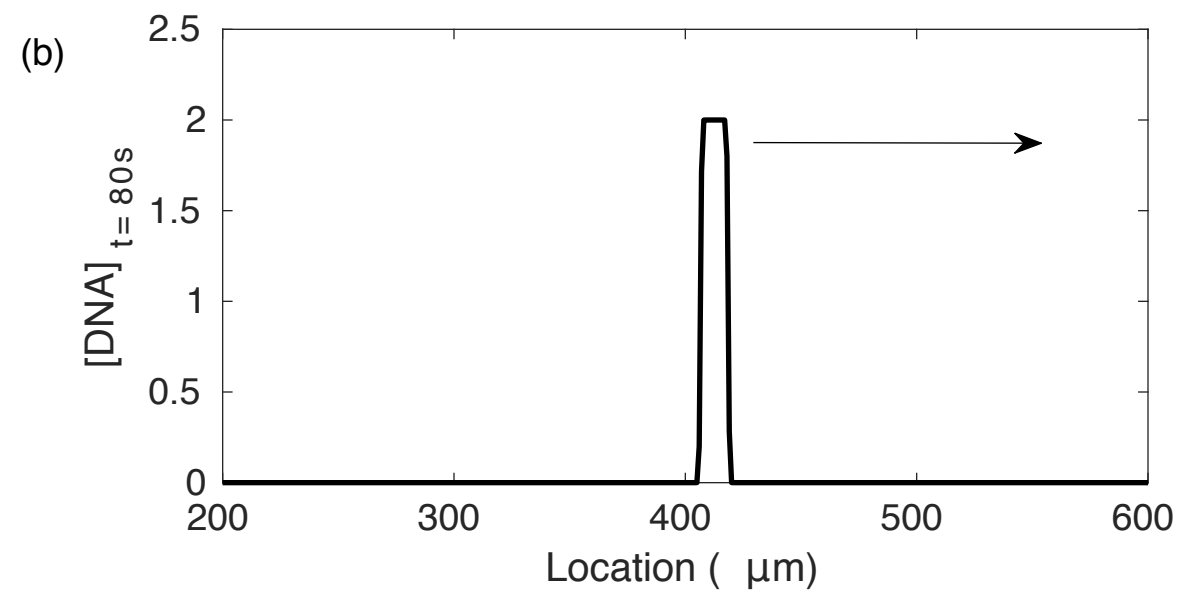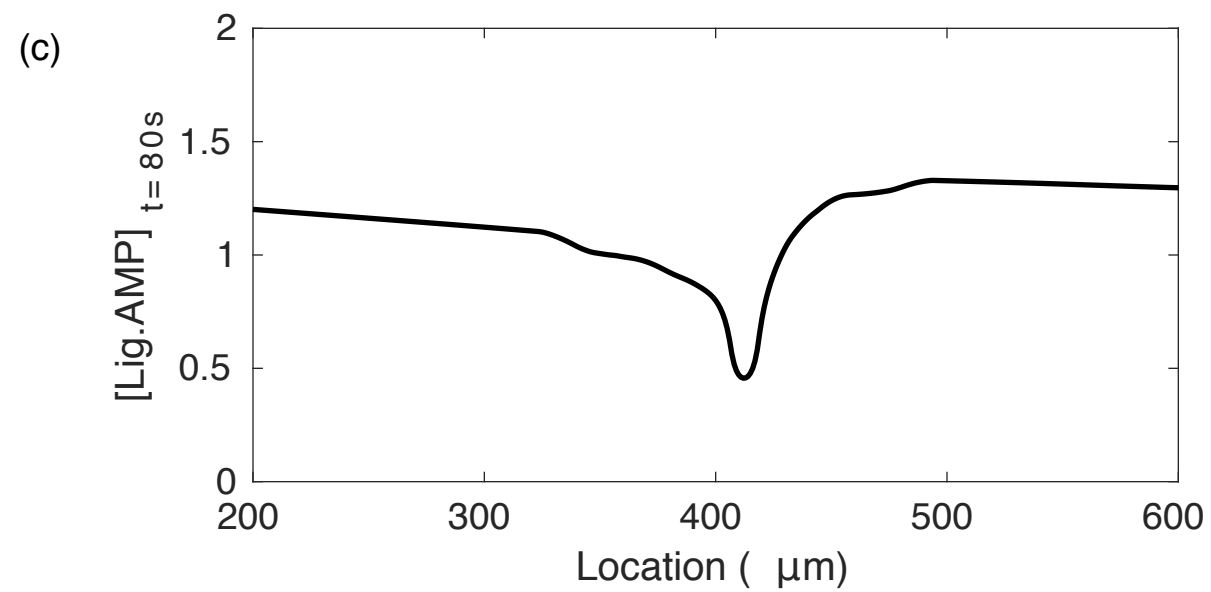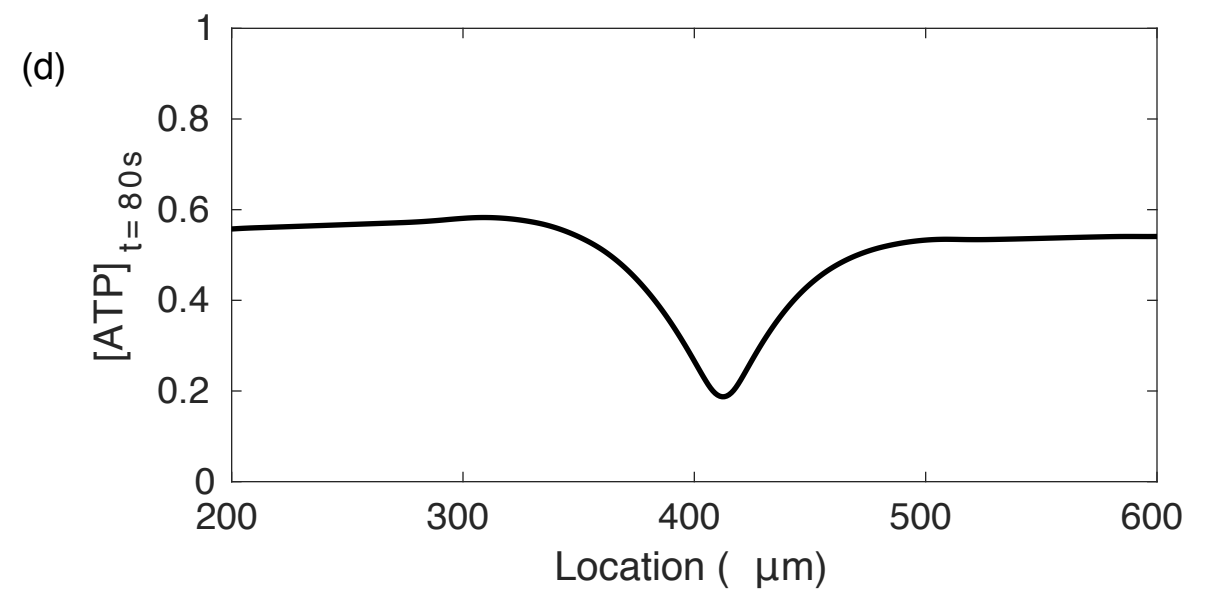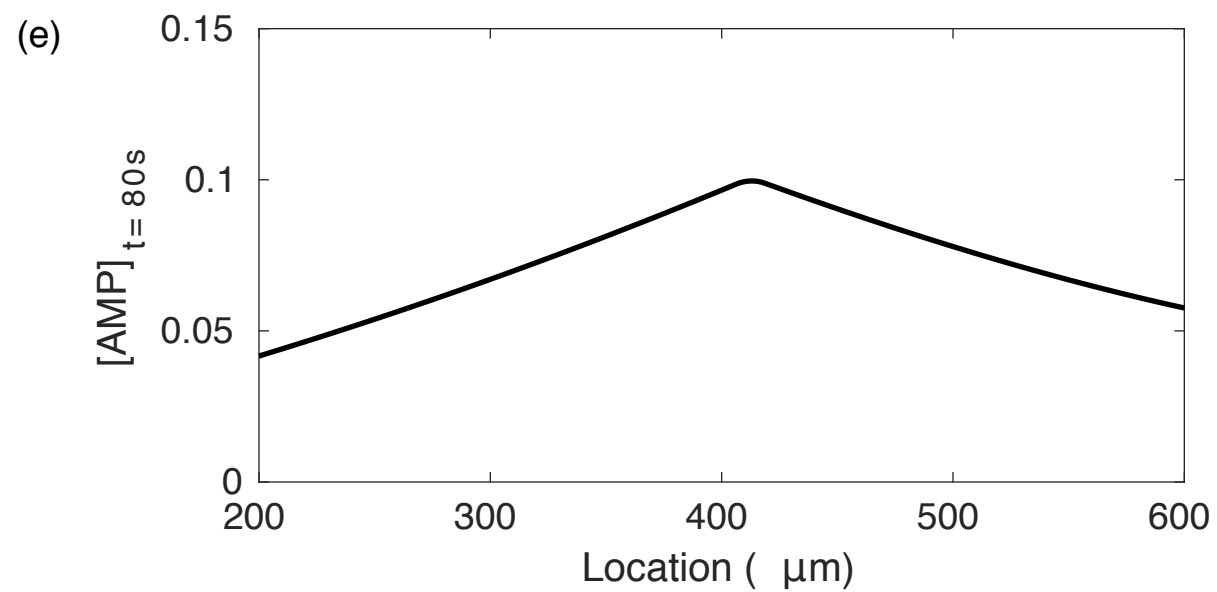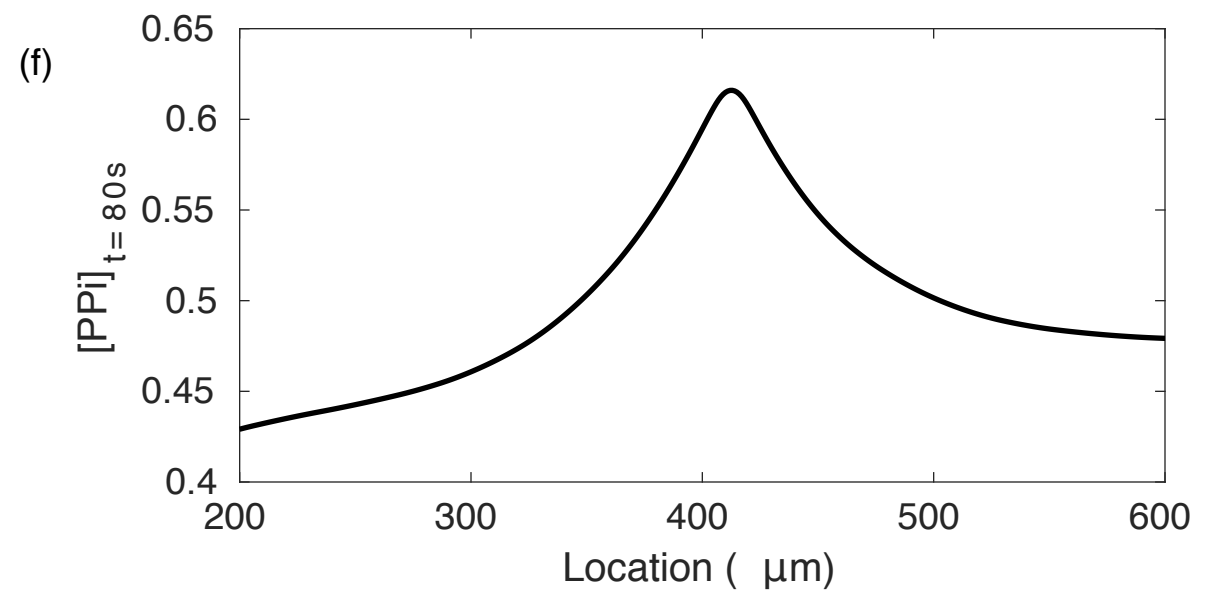

Supplement: Supplementary file 1 — LaTeX Supplementary File [file 41598_2018_28278_MOESM1_ESM.pdf]

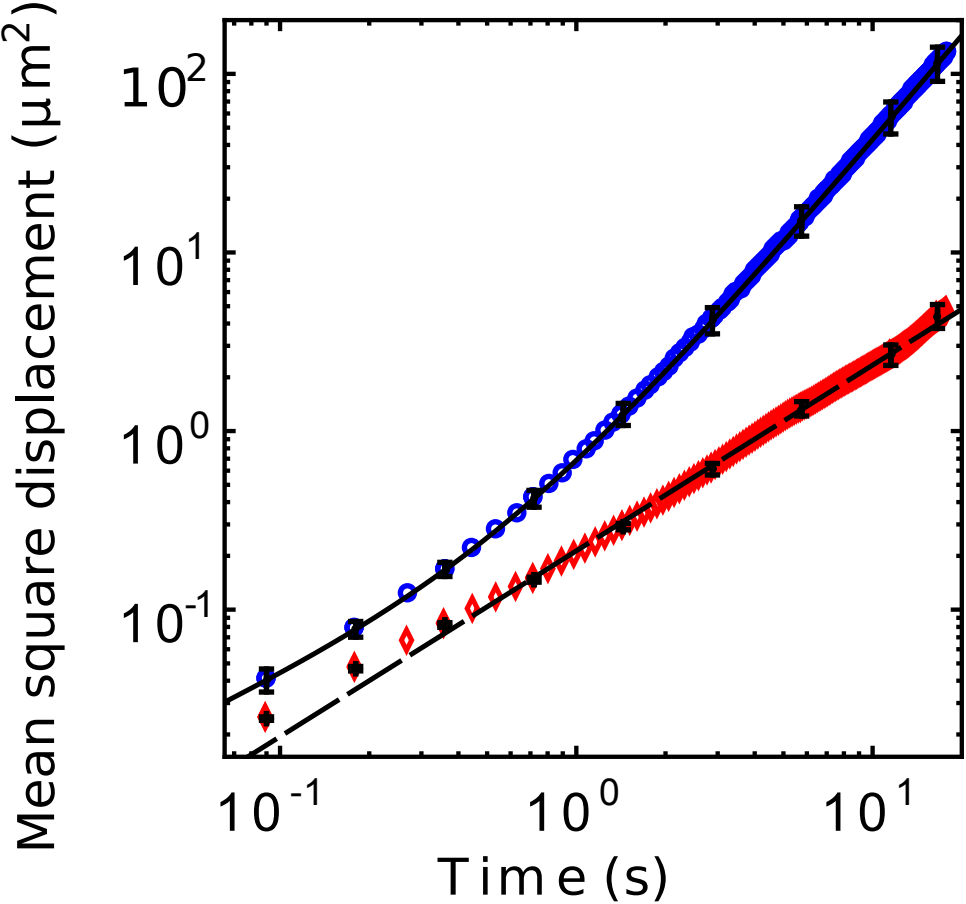

Supplement: Supplementary file 2 — LaTeX Supplementary File [file 41598_2018_28278_MOESM2_ESM.pdf]

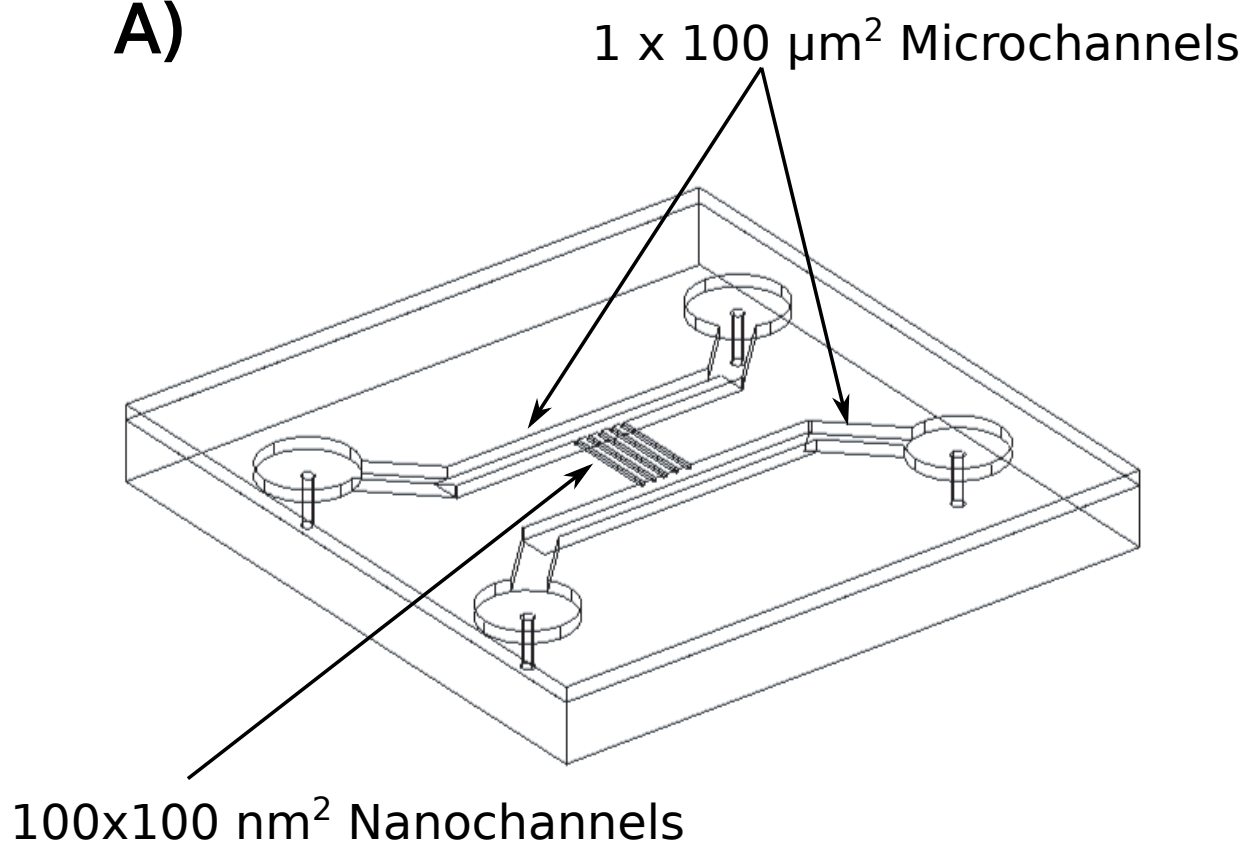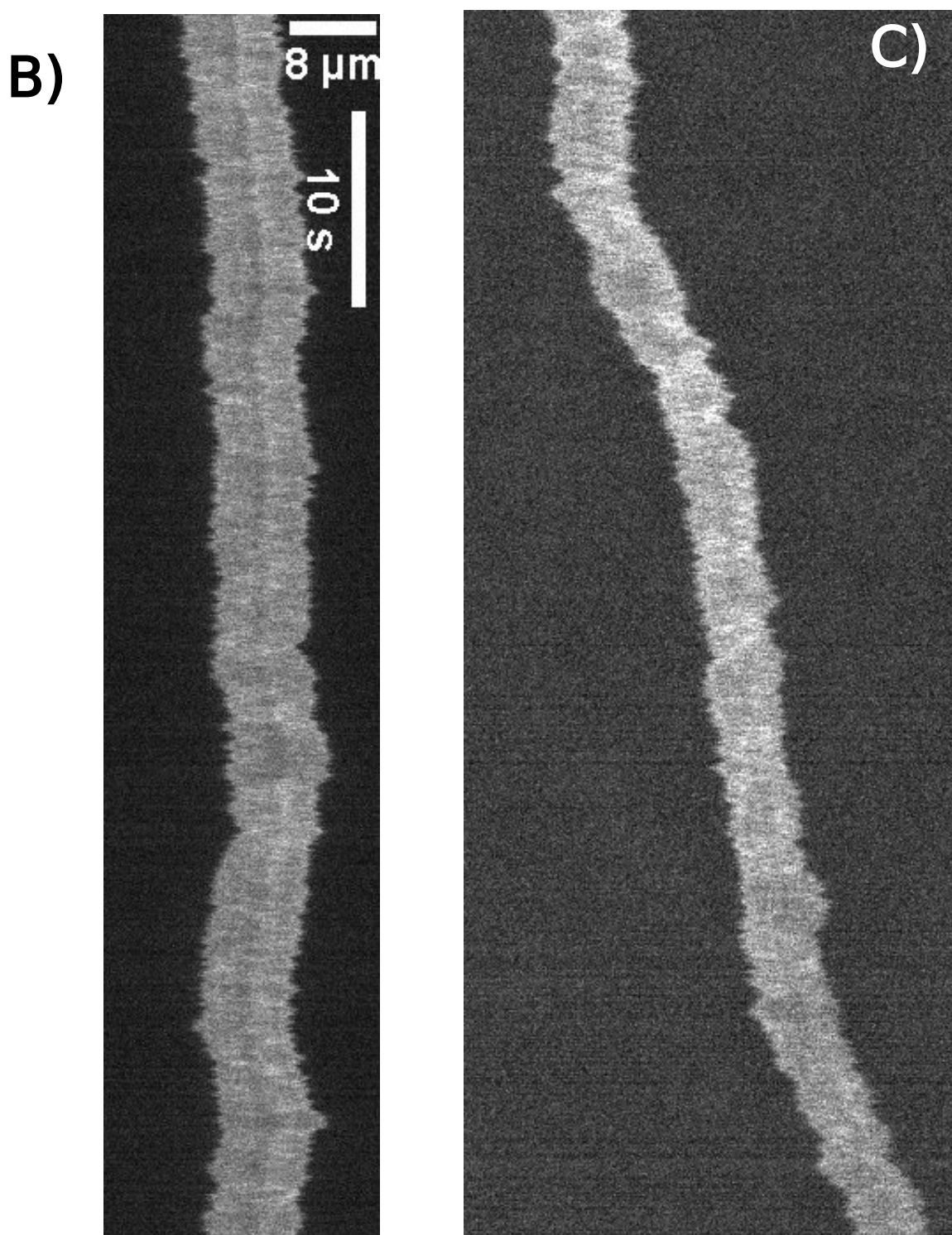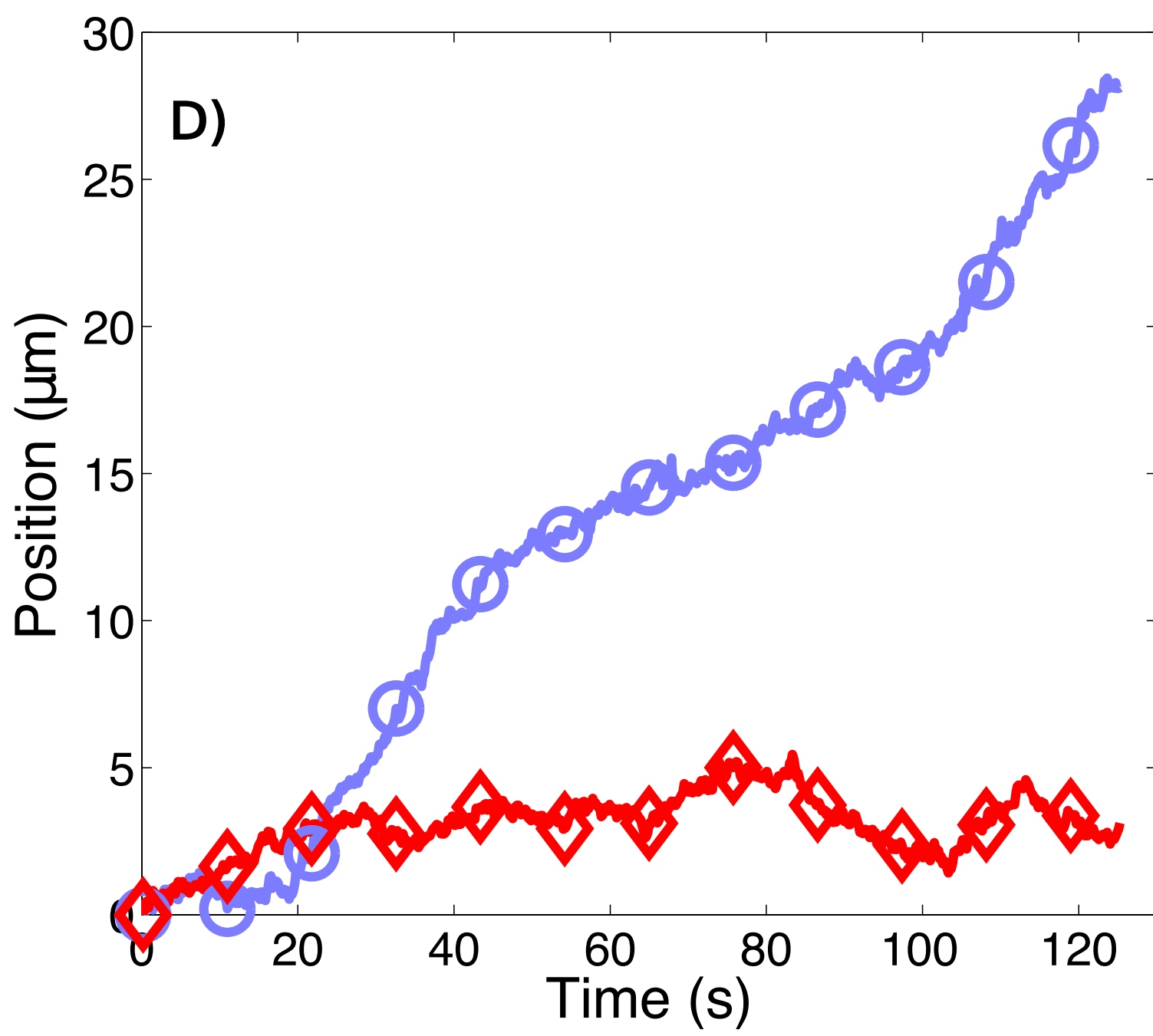

Supplement: Supplementary file 3 — LaTeX Supplementary File [file 41598_2018_28278_MOESM3_ESM.pdf]

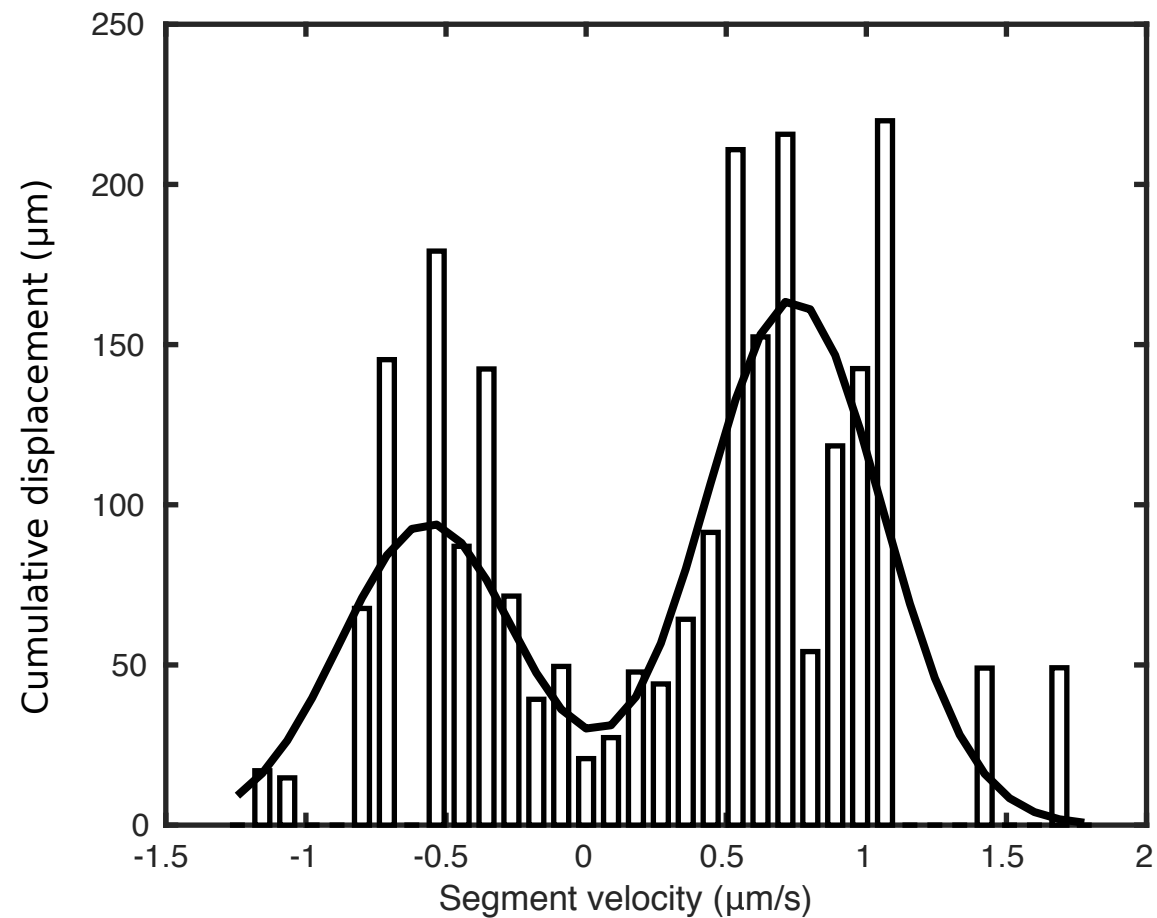

Supplement: Supplementary file 4 — LaTeX Supplementary File [file 41598_2018_28278_MOESM4_ESM.pdf]
