# Supplementary material for: Motor-like DNA motion due to an ATP-hydrolyzing protein under nanoconfinement
Source: Sci Rep. 2018 Jul 3;8:10036. doi: 10.1038/s41598-018-28278-0 (PMC6030079; doi:10.1038/s41598-018-28278-0)
Supplement: Supplementary file 5 — Supplementary Materials [file 41598_2018_28278_MOESM5_ESM.pdf]

Supplementary material for: Motor-like DNA motion due to an ATP-hydrolyzing protein under nanoconfinement.

Maedeh Roushan, Zubair Azad, Saeid Movahed, Paul D. Ray, Gideon I. Livshits, Shuang Fang Lim, Keith R. Weninger, Robert Riehn*

E-mail: rriehn@ncsu.edu

A. Device layout

The device follows the commonly used H-shaped layout in which two microchannels are bridged by nanochannels. We fabricate the device in fused silica using electron beam lithography, optical lithography, and reactive ion etching. Access holes are drilled through the substrate. The device measures 25×25 mm^2, and access holes are on a 12.5 mm grid. A fused silica coverslip is bonded to the device (Fig. 1A in the manuscript).

B. Effective concentration of T4 DNA ligase

In an earlier publication we discussed the effective concentration of DNA for different vendors.[1] Since this is equally relevant for this paper, we reproduce the exact section here:

We chose T4 DNA ligase supplied by Roche Diagnostics (1 $U_{\mathrm{Roche}}/\mu l$), since this vendor does not supplement their product with BSA. The latter would interfere with the interpretation of our AFM measurements. The T4 DNA ligase stock solution was expected to have a concentration of 6 μM, while we measured a concentration of 10.4 μM via absorption spectroscopy. We also observed

that T4 DNA ligase is predominantly in a multimeric state using dynamics light scattering (DLS), with some large aggregates, possibly due to the fact that this vendor does not add BSA. We thus believe the concentration of active T4 DNA ligase molecules is considerably lower. Using Roches units, the final T4 DNA ligase concentration is 5 U_{Roche}*/ml.*

The majority of recent publications in the field use T4 DNA ligase supplied by New England Biolabs, which contains BSA. Using the same unit definition as New England Biolabs, the final concentration of T4 DNA ligase is in the range of 500 U_{NEB}*/ml to 1000* U_{NEB}*/ml (1* U_{NEB}*/ml = 0.02 nM[2]). This compares to 250* U_{NEB}*/ml used by Widom and Cloutier,[3,4] 1* U_{NEB}*/ml used by low-concentration studies such as Du et al.,[5] and a few* 10^4 U_{NEB}*/ml by Yuan et al.[2] We verified that nanochannel measurements with an equivalent concentration of T4 DNA ligase from both New England Biolabs and Roche gave similar results.*

C. Extension of DNA molecules along nanochannels

In the main manuscript we state that DNA is extended inside nanochannels, and that DNA contracts somewhat when T4 DNA ligase, ATP, Mg^{2+}, and EDTA are added. In supplementary Fig. 1 we show the distribution of lengths for buffers both with and without all co-factors. DNA in Tris-borate buffer without proteins or co-factors showed an extension consistent with expectations, with only one sporadic outlier. The main peak of the histogram for DNA with T4 DNA ligase and all co-factors contracted by about 1/4, in line with expectations. However, 1/4 of all molecules were not part of the main peak, but rather a satellite shorter than the main peak. We attribute this fact to photodamage during the more extended bench time that this sample experiences before it reaches the device. This would be indication that we should anticipate a considerable number of nicks on the sample at the time of introduction into the device.[6]

We also included the length distribution for a data set obtained using "human genomic DNA", sold by Roche. The extension was about 2/3 of that for λ-DNA at the same condition.

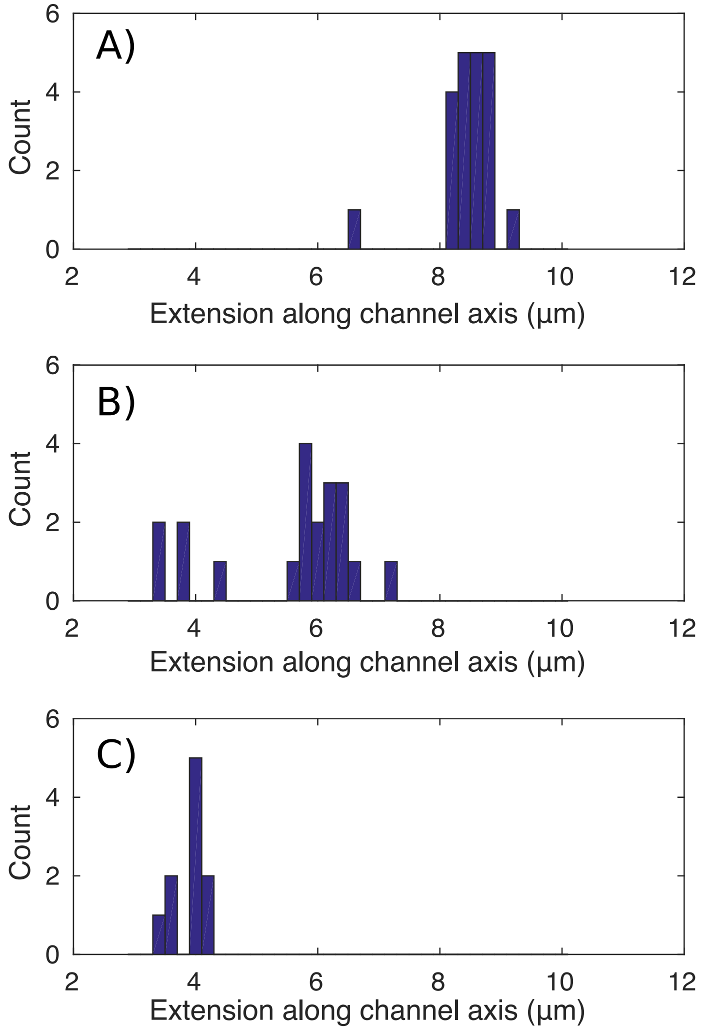

Figure 1: Extensions of DNA along channel axis. A) λ-DNA in absence of T4 DNA ligase, ATP, and Mg^{2+}. B) λ-DNA in presence of the full system of T4 DNA ligase, ATP, and Mg^{2+} and EDTA. C) Human genomic DNA sample with T4 DNA ligase, ATP, and Mg^{2+} and EDTA.

D. Supplementary data for mean square displacement analysis

Table 1 of the main manuscript we list $\left\langle \Delta x(\Delta t)^2 \right\rangle$ relationships for a range of control experiments that test motion in buffers containing subsets of the full system for T4 DNA ligase, as well as E. coli DNA ligase and MutS. The corresponding mean square displacement (MSD) versus time graphs are shown in the following sections. They are entirely analogous to the plots in Fig. 2 of the main manuscript. For consistency, axes are held constant for all plots.

D.i. Supplementary data for T4 DNA ligase

The MSD versus time graphs for the T4 DNA ligase data set that are listed in main manuscript Tab. 1 but not shown in main manuscript Fig. 2 are presented in supplementary Fig. 2. In the table, the classification of "No" or "Yes" in the "Drift" column is based on whether a single power law with $\alpha \leq 1.3$ was sufficient to describe the data, or whether two power laws with one power law with $\alpha > 1.3$ was required.

We further include a graph for the "human genomic DNA" from Roche in presence of T4 DNA ligase, ATP, Mg^{2+} and EDTA. It shows the same superdiffusive motion with $\left\langle \Delta x(\Delta t)^2 \right\rangle = 0.44\,\mu m^2 \left(\Delta t \frac{1}{s}\right)^{0.52} + 0.59\,\mu m^2 \left(\Delta t \frac{1}{s}\right)^{2.09}$. The exponent for the directed component is thus very close to that of the λ-DNA sample, and the speed of translocation is within the error bars for a $10\,s$ observation base.

D.ii. Supplementary data for MutS

In the manuscript we assert that MutS causes the same super-diffusive drift as T4 DNA ligase under the same conditions. For MutS, we additionally tested activity under γ-ATP, where we found minimal superdiffusive activity with an MSD more than one order of magnitude lower than in presence of ATP. The data is summarized in supplementary Fig. 3.

D.iii. Supplementary data for E. coli DNA ligase

In the manuscript we assert that E. coli DNA ligase causes the same super-diffusive drift as T4 DNA ligase, but only in presence of NAD^+ and not ATP. In supplementary Fig. 4 we present the relevant MSD plots. Both graphs are best described by a two power law fit. For NAD^+ , the faster of the two components is consistent with drift with a constant velocity. The graph with ATP is consistent with classical diffusion at long times (the slope in the log-log plot should be equal in both graphs if there was drift in both).

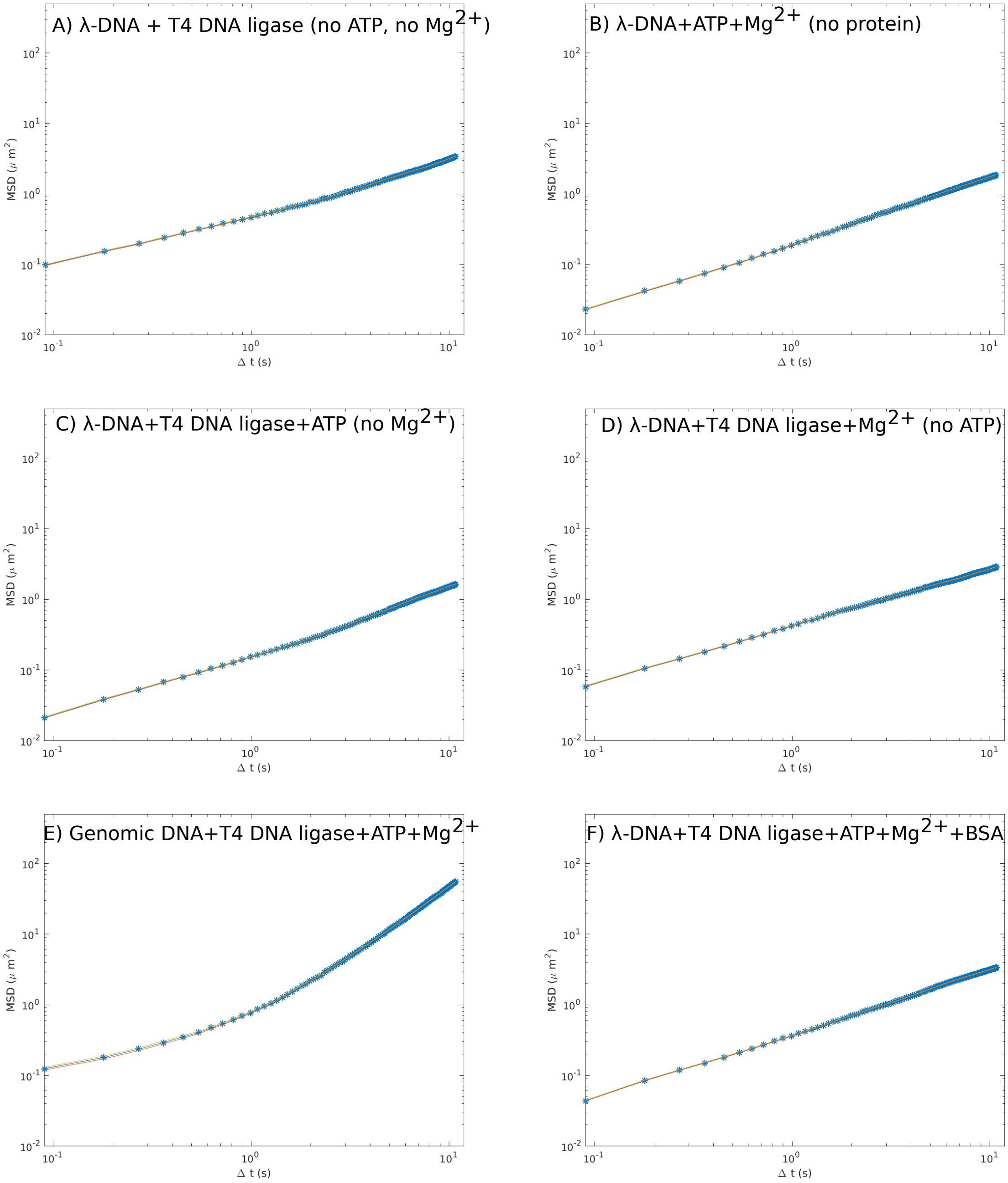

Figure 2: Supplementary data for drift under influence of T4 DNA ligase. A) λ-DNA and T4 ligase without co-factors. B) λ-DNA without T4 DNA ligase, but with cofactors ATP, Mg^{2+} and EDTA present. C) λ-DNA with T4 DNA ligase in presence of ATP, but no Mg^{2+}. D) λ-DNA and T4 DNA ligase in presence of Mg^{2+} and EDTA, but no ATP. E) Genomic human DNA in presence of the full system of T4 DNA ligase, ATP, Mg^{2+}, and EDTA. F) λ-DNA in presence of the full system of T4 DNA ligase, ATP, Mg^{2+}, and EDTA with 0.6 mg/ml BSA.

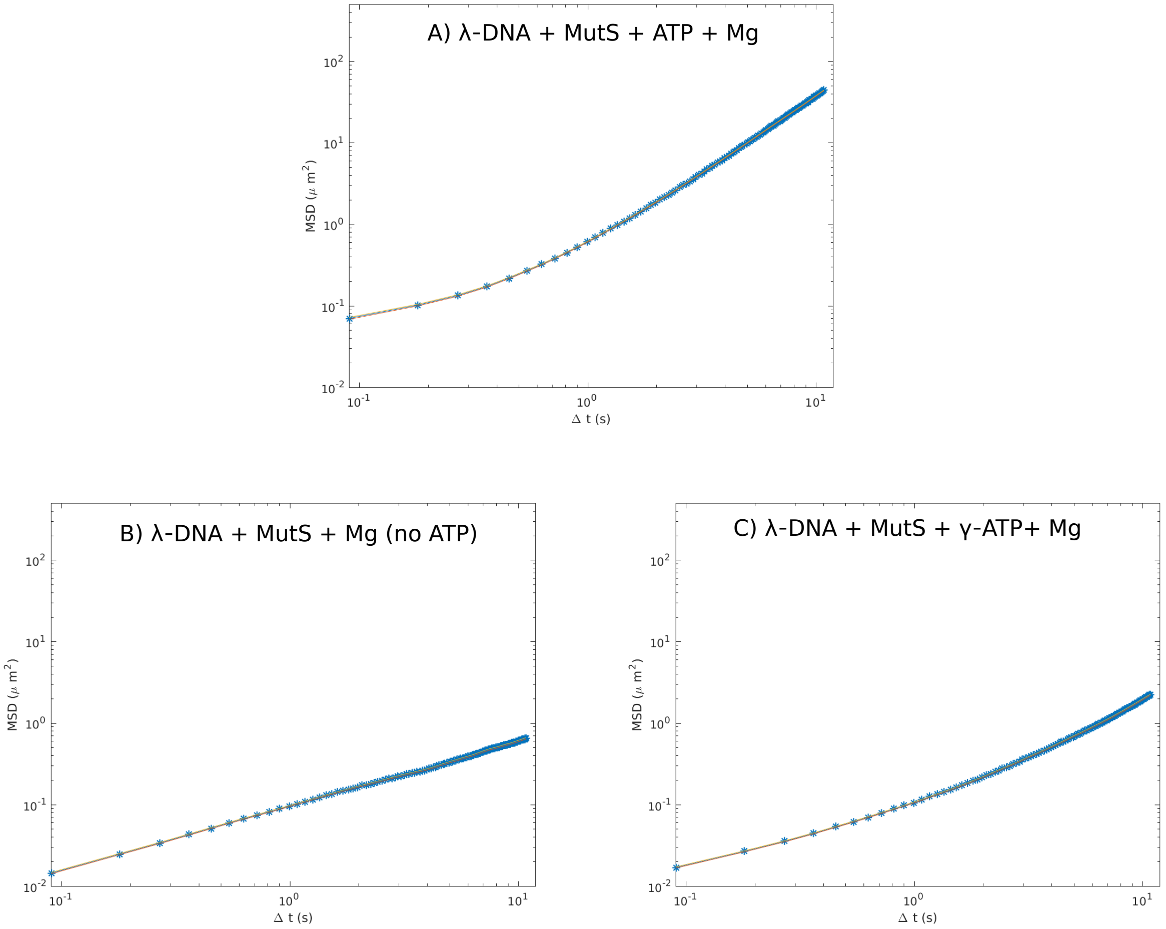

Figure 3: Supplementary data for drift under influence of MutS. A) λ-DNA in presence of MutS, ATP, Mg^{2+}, and EDTA. B) λ-DNA in presence of MutS, Mg^{2+} and EDTA, but no ATP. C) λ-DNA in presence of MutS, γ-ATP, Mg^{2+} and EDTA.

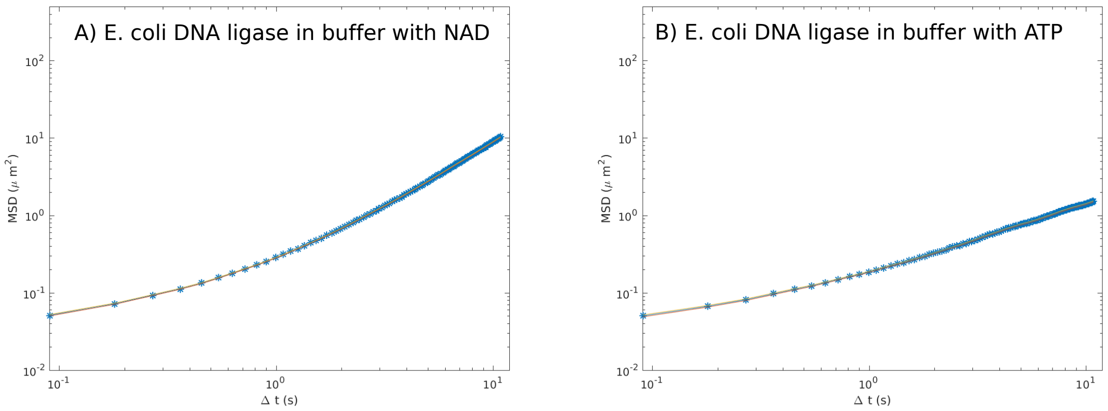

Figure 4: Supplementary data for displacement under influence of E. coli DNA ligase. A) λ-DNA in presence of E. coli DNA ligase and NAD^+. B) λ-DNA in presence of E. coli DNA ligase and ATP.

E. Decomposition of motion into stepwise constant motion sections

Through visual inspections of the kymograph in Fig. 1C of the main manuscript it is apparent that the DNA in presence of T4 DNA ligase moves with a step-wise constant drift speed. To arrive at Fig. 4 of the main manuscript, we segmented the motion into regions of approximately constant drift by fitting a piecewise linear function.

The number of segments was determined in the following fashion: We assumed that no directed motion is present for DNA without any protein, and that the error fitting a straight line to the position versus Δt graph in that purely diffusive data should be the lower bound for the uncertainty of fitting linear segments to DNA undergoing super-diffusive motion. The average mean squared error of a linear fit to a position versus time plot for such a molecule is $0.2\,\mu m^2$ over $20\,s$. We started analyzing position versus time curves for DNA undergoing diffusive motion by attempting fitting with a single linear segment. If the mean squared error was larger than $0.2\,\mu m^2$, the fit was attempted using two segments, and if that still resulted in a mean squared error larger than $0.2\,\mu m^2$ yet another segment was added. This procedure continued until the quality of fit criterion was met. The fitting is illustrated in supplementary Fig. 5. During fitting, the segments were parametrized by varying the transition points between the different segments. Both the mean drift velocity of the segment and the time duration of the segment were extracted. The product of both

gives the displacement distance during a segment. The distribution of dislocation distances versus translocation speeds over all molecules for the active enzymatic system is presented in Fig. 4 of the main manuscript.

We note here an observation that possibly is relevant to the contribution of wall interactions to the directed motion process. In supplementary Fig. 6 we show that the mean duration of a segment is on the order of 10 s. Note that this could be by design since the fit quality criterion for a segment was derived over a similar time span. However, the decision to plot main manuscript Fig. 2 to about 15 s was based both the apparent length of linear segments on first visual inspection, as well as the thinning statistics and diverging errors beyond that point. During this mean segment time of 10 s, DNA translocates by about 7 μm (main manuscript Figs. 2 and 4). Interestingly, this distance coincides with the DNA extension in supplementary Fig. 1. This could point toward a role of wall-bound T4 DNA ligase, since an active site bound to the wall would contribute to the motion for as long as it is in contact with the DNA. However, it could also point to the retarding effect of patches along the channel.

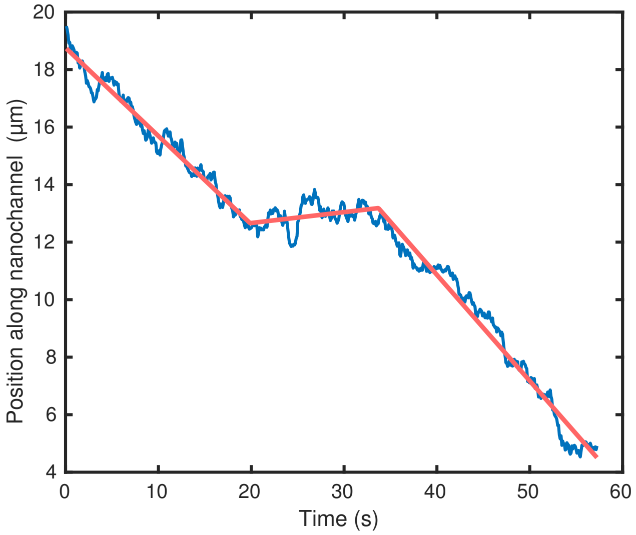

Figure 5: Decomposition of motion into segments. Same conditions as Fig. 1C of the main manuscript.

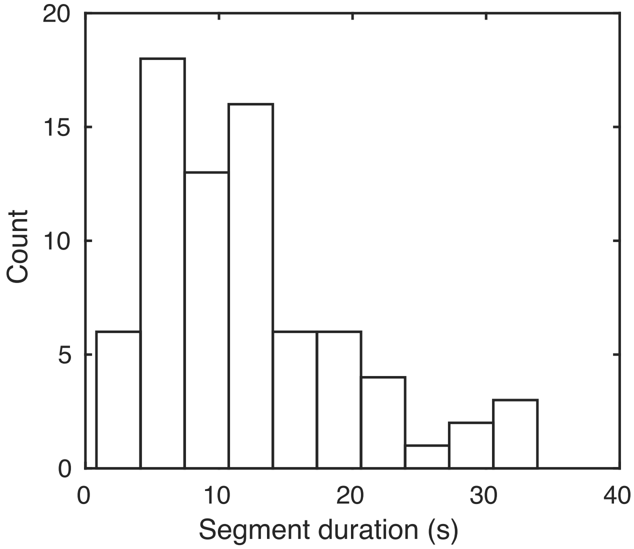

Figure 6: Histogram of segment durations in the data set contributing to Fig. 4 of the main manuscript.

F. Persistence of DNA transport direction under driving

As stated in the manuscript, the direction of the DNA transport did not change when the molecule was left unperturbed. We also tested whether this imprinting of transport direction is robust to moving the molecule along the nanochannel by applying a pressure-driven flow. We anticipate that DNA moves *together* with the liquid through the channel. Direct mechanical wall friction is typically thought to be unimportant, and all friction is mediated by hydrodynamics.[7] In supplementary Fig. 7 we perturbed a drifting DNA molecule (T4 DNA ligase + all cofactors) by applying a pressure gradient in the direction opposite to the direction of drift, and show that the molecule continues the motion in the original direction after the externally imposed flow is stopped. This implies that the "memory" of the drift direction has traveled with the DNA, and cannot be a property of the channel walls.

G. Estimation of nick density

According to Åkerman and Tuite,[6] the breakup of YOYO-1 stained DNA under light can be attributed to an accumulation of single-stranded nicks. When two nicks appear on opposite strands of the double helix with a sufficiently short nick to nick distance, the free energy of hybridization

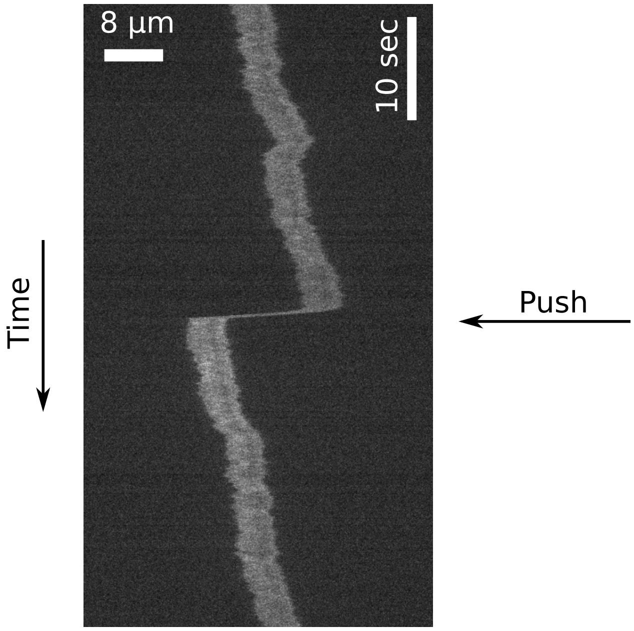

Figure 7: Drift of molecule with intermittent hydrodynamic push in direction opposite to travel in presence of T4 DNA ligase, ATP, Mg^{2+} and EDTA.

of the overlap can be overcome by thermal fluctuations and a double-stranded break becomes apparent. In this publication, we recorded each molecule to the point in time at which the first break in λ-DNA becomes visible. This is on the order of 45 s under the light intensity here.

While models of varying sophistication are available in the literature for the hybridization stability of DNA oligomers, we here use the simplistic estimate that at room temperature the longest stable stretch is 8 basepairs long,[8] which coincides with the footprint of up to two YOYO-1 dye molecules. λ-DNA then is 6000 of these 8-bp units long, and we have one of these 6000 units with a configuration where two nicks are on opposing strands. A probability calculation with two strands and two dye molecules per 8-bp unit shows that at the time of breaking, the λ-DNA molecule carries about 220 nicks. Probably DNA already carries some nicks when it is introduced into the nanochannel. However, the estimate sets an upper limit to about 5 new nicks per second.

H. Determination of DNA drift velocity within an ionic gradient under nanoconfinement

The determination of the drift direction of DNA when placed in a ionic strength gradient proceeds through independently measuring the DNA drift velocity and the flow velocity of the liquid column. We follow, conceptually, the work by Lee et al.[9] We fill one microchannel of our device with 1/8× TBE, and the other one with 2× TBE. The ionic strengths of TBE buffers at these formulations are well characterized, with $6.26\times10^{-2}M$ at 2× TBE and $2.86\times10^{-3}M$ at 0.1× TBE, and nearly linear scaling of ionic strength and buffer concentration.[10] The buffer in the high ionic strength microchannel contains Sulforrhodamine 640 (S640). We apply an external pressure either from the high or low ionic strength side, and record the dynamic time-dependent response of the fluorescent dye profile. The switch-over from no-pressure to constant pressure occurs on the order of 10s of milliseconds, and the change in dye profiles occurs on the order of 10s of seconds.

In Supplementary Fig. 8A, we show the kymograph of the fluorescent dye concentration along the channel axis for a sequence for applied forward/backward pressures. The S640 dye was added to the left channel, which also carries the high ionic strength buffer. Lee et al. extract the flow velocity from the steady-state concentration profile of the dye by fitting with the steady-state solution to the drift-diusion equation.[9] We extended their framework to a time dependent treatment, and show in Supplementary Figs. 8B,C that the time-dependent profiles are well-fit both for pressure applied on the high and the low ionic strength side. We concur with Lee[9] that liquid flows from the high ionic strength to the low ionic strength side.

With the flow velocity of the buffer medium established, we then placed DNA molecules into the nanochannel. In supplementary Fig. 9 we show a series of experiments with different static pressure differences between left and right channels. The velocities are such that positive numbers indicate motion from the left (high ionic strength) to the right (low ionic strength). In all cases, the DNA molecule had an relative motion to the liquid that drives it from the low ionic strength side to the high ionic strength side.

A complete description of this work is the topic of an upcoming manuscript.

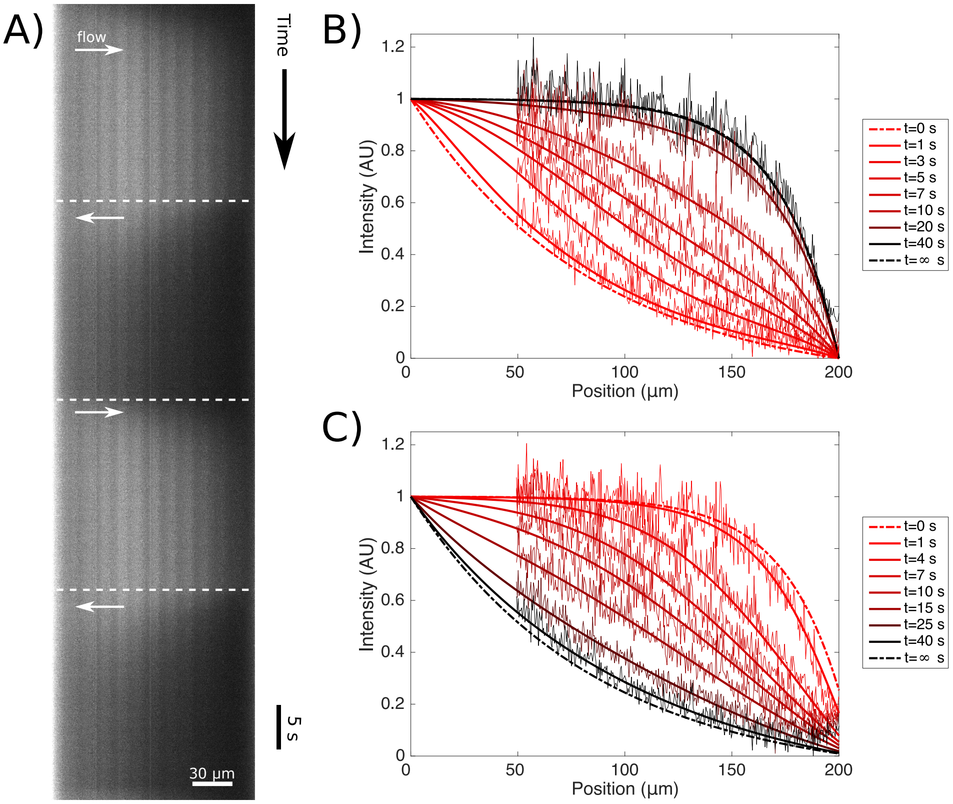

Figure 8: Time-dependent fluorescence intensity of fluorescent tracer in ionic strength gradient with high ionic strength on left and low ionic strength on right. A) shows kymographs of alternating pressure application of flow from left and right. B) shows the time-dependent change from flow right to left to flowing left to right. C) shows the time-dependent change from flow left to right to right to left.

I. Numerical Simulation of DNA drift in a nanochannel

To show the self-consistency of the hypothesis that self-maintaining concentration gradients give rise to the drift, we developed a one-dimensional drift-diffusion numerical code that is coupled to the enzymatic pathway of T4 DNA ligase. Using this code, the motion of DNA has been modeled with and without the enzymatic reaction of the T4 DNA ligase (Fig. 3 of the main manuscript).

The simulation was carried out over a quasi-infinite, one-dimensional channel (800 μm long). We assume that the on time scales beyond a few milliseconds, the solution is electrically neutral everywhere, and that the background buffer is homogeneous. We consider the background to consist of Tris-Borate, $MgCl_2$, and EDTA. Channel ends were assumed to obey a Neumann boundary condition. Note that we do not have access to all applicable reaction and diffusion rates that are

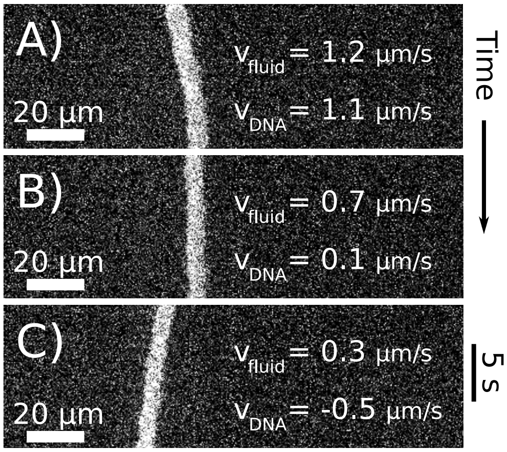

Figure 9: Motion of DNA in ionic strength gradient with high ionic strength on left and low ionic strength on right. Kymographs show a representative molecule for three different imposed pressure differences between left and right channel. Indicated velocities read positive when going left to right. Rhodamine tracer dyes were recorded on a different spectral channel and velocities extracted as illustrated above.

used in the model. Rather, we used rates that estimated to at least an order of magnitude, or chosen such that the simulation gave results consistent with experiment. We do not claim a quantitative treatment, but rather a numerical treatment that discovers qualitative traits. We anticipate that the qualitative traits are robust, while any quantitative results would rescale with a change in the numerical reaction and diffusion parameters. We do not non-dimensionalize results.

For each of the critical species (T4 DNA ligase, adenylated T4 DNA ligase, DNA, DNA bound to adenylated T4 DNA ligase, ATP, PPi and AMP) of the proposed model, a diffusive mass transfer equation should be solved to find the transport and development of the concentration of each ionic species c_i in a nanochannel

$$\frac{\partial c_i}{\partial t} = D_i \frac{\partial^2 c_i}{\partial x^2} + R_i \ . \tag{1}$$

In this equation, c_i and D_i are the concentrations and diffusion coefficients of species i, t represents time and R_i is the source term of the species within the enzymatic reaction cascade generally proposed for T4 DNA ligase. The following reactions were implemented: T4L + ATP $\rightleftharpoons$ T4L.A + PPi, T4L.A + DNA $\rightleftharpoons$ T4L.A.DNA, T4L.A.DNA $\rightleftharpoons$ T4L + AMP + DNA. In these reactions, T4L is T4 DNA ligase, T4L.A is adenylated T4 DNA ligase, and T4L·A·DNA is adeny-

lated T4 DNA ligase bound to DNA. We assume sufficient photo-induced nicks being created by the process of fluorescence imaging such that target sites for the enzyme are dense along DNA, and thus we did not track the distribution of nicks. All reactions follow a standard mass-action law such that (while writing $c_{\text{Species}} = [Species]$)

$$\begin{aligned}
R_{\text{ATP}} &= -r_1[T4L][ATP] + r_2[T4L.A][PPi] && (2)\\
R_{\text{PPi}} &= r_1[T4L][ATP] - r_2[T4L.A][PPi] && (3)\\
R_{\text{T4L}\cdot\text{A}} &= r_1[T4L][ATP] - [T4L\cdot A]\,(r_2[PPi] + r_3[DNA]) + r_4[T4L\cdot A\cdot DNA] && (4)\\
R_{\text{T4L}\cdot\text{A}\cdot\text{DNA}} &= r_3[T4L\cdot A][DNA] - (r_4 + r_5)[T4L\cdot A\cdot DNA] + r_6[T4L][AMP][DNA] && (5)\\
R_{\text{AMP}} &= r_5[T4L.A\cdot DNA] - r_6[T4L][AMP][DNA] && (6)\\
R_{\text{T4L}} &= -[T4L]\,(r_1[ATP] + r_6[AMP][DNA]) + r_2[T4L\cdot A][PPi] + && (7)\\
&\quad r_5[T4L\cdot A\cdot DNA] && (8)
\end{aligned}$$

While we not have an analytic model for the drift of DNA in a nanochannel, we do know that DNA drifts towards regions of high ionic strength ($I = \frac{1}{2}\sum z_i c_i^2$) and thus should depend on the gradient of I. However, the overall drift velocity in higher strength buffers does not increase when compared to lower strength buffers. Following[11] *et al.* both of these conditions are met by a force density (per unit length of nanochannel occupied by DNA) of

$$f\left(I, \frac{\partial I}{\partial x}\right) \propto \frac{1}{I}\frac{\partial I}{\partial x} \propto \frac{\partial}{\partial x}\ln I \ . \qquad (9)$$

The force on the entire DNA molecule is determined by

$$F_{\text{DNA}} = \int_{\text{along DNA}} f\left(I, \frac{\partial I}{\partial x}\right) dx \ , \qquad (10)$$

and the displacement of the DNA molecule is derived as

$$\frac{\partial X_{\mathrm{CM}}}{\partial t} = \eta_{\mathrm{DNA}} F_{\mathrm{DNA}} \ , \tag{11}$$

where X_{CM} is the center of mass of the DNA molecule and η_{DNA} a mobility. The length (extension along channel) of DNA was fixed in our simulation, and the DNA concentration above was found from the center of mass and length. T4 DNA ligase bound to DNA was moved with DNA. The concentrations of ionic species where initialized as Gaussians whose centers of mass were offset relative to the center of mass of the DNA. For the drift model, we assumed that the phosphates in ATP are less completely deprotonated than the phospates in PPi and AMP. Thus, the ionic strength of the solution increases as ATP is hydrolyzed. The exact difference in degree of ionization is not required to understand the drift process itself.

The numerical results of the model are presented in Fig. 3 of the main manuscript. Fig.3(a) presents the movement of the center of mass of the DNA along the nanochannel. The DNA will be stationary in the absence of ATP-hydrolyzing and enzymatic chemical reaction. However, the ATP-hydrolyzing reaction will cause linear transport of the DNA in the system which is in good agreement with the experimental observations. For the purpose of this figure, we only plotted times beyond which the initial asymmetry in ion concentrations had completely dissipated in the case without enzymatic activity ($> 80s$). The simulations were repeated for three initial positions of the DNA ($390nm$, $400\,\mu m$ and $410\,\mu m$). The identical movement of the DNA in these three cases of study prove the stability of the numerical model.

Similarity of the experimental and the numerical results of the current study confirm the self-consistency of the proposed model for the motor-like DNA motion due to an ATP-hydrolyzing reaction, and thus strengthen our hypothesis that the effect is not specific to the limited set of proteins tested here, but should be observed in general.

Table 1: The assumed constants and parameters of the current study

Parameter	Description	Value
l	Length of the nanochannel	$800\,\mu m$
x_0	Initial position of the DNA	$400\,\mu m$
D_{ATP}	Diffusion coefficient of ATP	2×10^{-11} (m^2/s)
D_{AMP}	Diffusion coefficient of AMP	0.816×10^{-9} (m^2/s)
D_{PPi}	Diffusion coefficient of PPi	7.99×10^{-11} (m^2/s)
D_{AMP}	Diffusion coefficient of T4L	0.816×10^{-12} (m^2/s)
D_{PPi}	Diffusion coefficient of Lig.AMP	7.99×10^{-12} (m^2/s)

References

(1) Roushan, M.; Azad, Z.; Lim, S. F.; Wang, H.; Riehn, R. *Microchimica Acta* **2015**, *182*, 1561–1565.

(2) Yuan, C.; Lou, X. W.; Rhoades, E.; Chen, H.; Archer, L. A. *Nucleic Acids Research* **2007**, *35*, 5294–302.

(3) Cloutier, T. E.; Widom, J. *Proceedings of the National Academy of Sciences of the United States of America* **2005**, *102*, 3645–50.

(4) Cloutier, T. E.; Widom, J. *Molecular Cell* **2004**, *14*, 355–362.

(5) Du, Q.; Smith, C.; Shiffeldrim, N.; Vologodskaia, M.; Vologodskii, A. *Proceedings of the National Academy of Sciences of the United States of America* **2005**, *102*, 5397–402.

(6) Åkerman, B and Tuite, E, *Nucleic Acids Research* **1996**, *24*, 1080–90.

(7) Tree, D. R.; Wang, Y.; Dorfman, K. D. *Biomicrofluidics* **2013**, *7*, 054118.

(8) Wallace, R. B.; Shaffer, J.; Murphy, R.; Bonner, J.; Hirose, T.; Itakura, K. *Nucleic Acids Research* **1979**, *6*, 3543–3558.

(9) Lee, C.; Cottin-Bizonne, C.; Biance, A. L.; Joseph, P.; Bocquet, L.; Ybert, C. *Physical Review Letters* **2014**, *112*, 1–5.

(10) Hsieh, C.-C.; Balducci, A.; Doyle, P. S. *Nano Letters* **2008**, *8*, 1683–8.

(11) Palacci, J.; Abécassis, B.; Cottin-Bizonne, C.; Ybert, C.; Bocquet, L. *Physical Review Letters* **2010**, *104*, 1–4.
